# Attitudes and Acceptance of Oral and Parenteral HIV Preexposure Prophylaxis among Potential User Groups: A Multinational Study

**DOI:** 10.1371/journal.pone.0028238

**Published:** 2012-01-11

**Authors:** Andreas B. Eisingerich, Ana Wheelock, Gabriela B. Gomez, Geoffrey P. Garnett, Mark R. Dybul, Peter K. Piot

**Affiliations:** 1 Imperial College Business School, Imperial College London, London, United Kingdom; 2 Faculty of Medicine, Imperial College London, London, United Kingdom; 3 Amsterdam Institute for Global Health and Development, Amsterdam, The Netherlands; 4 Department of Infectious Disease Epidemiology, Imperial College London, London, United Kingdom; 5 Georgetown O'Neill Institute for National and Global Health Law, Georgetown University, Washington, D.C., United States of America; 6 London School of Hygiene and Tropical Medicine, London, United Kingdom; Burnet Institute, Australia

## Abstract

**Background:**

The use of antiviral medications by HIV negative people to prevent acquisition of HIV or pre-exposure prophylaxis (PrEP) has shown promising results in recent trials. To understand the potential impact of PrEP for HIV prevention, in addition to efficacy data, we need to understand both the acceptability of PrEP among members of potential user groups and the factors likely to determine uptake.

**Methods and findings:**

Surveys of willingness to use PrEP products were conducted with 1,790 members of potential user groups (FSWs, MSM, IDUs, SDCs and young women) in seven countries: Peru, Ukraine, India, Kenya, Botswana, Uganda and South Africa. Analyses of variance were used to assess levels of acceptance across different user groups and countries. Conjoint analysis was used to examine the attitudes and preferences towards hypothetical and known attributes of PrEP programs and medications. Overall, members of potential user groups were willing to consider taking PrEP (61% reported that they would definitely use PrEP). Current results demonstrate that key user groups in different countries perceived PrEP as giving them new possibilities in their lives and would consider using it as soon as it becomes available. These results were maintained when subjects were reminded of potential side effects, the need to combine condom use with PrEP, and for regular HIV testing. Across populations, route of administration was considered the most important attribute of the presented alternatives.

**Conclusions:**

Despite multiple conceivable barriers, there was a general willingness to adopt PrEP in key populations, which suggests that if efficacious and affordable, it could be a useful tool in HIV prevention. There would be a willingness to experience inconvenience and expense at the levels included in the survey. The results suggest that delivery in a long lasting injection would be a good target in drug development.

## Introduction

HIV remains a significant global health problem, with an estimated 2.6 million people newly infected in 2009, challenging both national and international decision makers to identify effective prevention interventions. Better access to treatment and new prevention strategies are urgently needed to control the spread of the virus [1].

The landscape of HIV prevention has been dramatically altered by recent trials of antiretroviral based prevention methods. Early treatment of those with HIV significantly reduced the risk of transmission to uninfected partners by 96% in the HPTN-052 trial, which was stopped early due to efficacy [2]. Another and potentially complementary approach is preexposure prophylaxis (PrEP), the use of antiretroviral medications to reduce the risk of HIV infection in people who are HIV negative. In the CAPRISA 004 trial, a tenofovir 1% vaginal gel reduced HIV infection rates by 39% [3]. The IPrEX study, a trial of oral dosing, showed that a daily dose of Truvada, an antiretroviral drug combination of tenofovir disoproxil fumarate and emtricitabine, reduced the risk of HIV infection by an average of 44% in HIV negative men and transgender women who have sex with men (MSM) [4]. The CDC TDF2 extended trial in Botswana found that a once-daily tablet of Truvada reduced the risk of acquiring HIV by an average of 63% in HIV negative heterosexual men and women [5]. Consistently, the Partners PrEP study in Kenya and Uganda showed that two different antiretroviral regimes significantly prevented HIV transmission among serodiscordant couples. 62% and 73% fewer HIV infections were observed in the tenofovir and Truvada arms of the trial, respectively, compared to those participants who received placebo [5]. Conversely, the FEM-PrEP and VOICE studies testing daily oral Truvada and tenofovir, respectively, among women, were stopped early for futility [6], [7]. Researchers are conducting additional analyses to explore what drove the observed lack of effect.

Further evidence from ongoing and planned trials of oral, topical and parenteral PrEP among different key populations at higher risk will be needed before the most effective strategy for antiretroviral (ARV) based prevention can be established. In the meantime, U.S. Centers for Disease Control and Prevention (CDC) has released interim guidance for the use of PrEP in MSM populations [8]. Guidance for PrEP use in other populations and countries may follow; but this would be against a backdrop of limited resources, competing priorities, and in some cases, cultural, religious, and legal barriers [9]. Countries also face the challenge of identifying whether to integrate PrEP as part of combination prevention and therefore may need to start preparing for an eventual PrEP implementation which is safe, efficacious, and affordable, and importantly, in tune with the needs and concerns of potential users.

An important aspect of access to and impact of any new treatment or prevention measure is whether it is adopted by potential users [10]. Yet most research efforts to date have focused on clinical aspects of PrEP and little attention has been paid to potential users' willingness to take it. Although there are a number of acceptability studies on vaginal gels [11], [12], [13], few studies have addressed other existing and potential routes of administration of PrEP. Four studies have examined the knowledge and off-label use of oral PrEP among MSM in the United States [14], [15], [16], [17] and two assessed its acceptability: one among female participants of a PrEP clinical trial in Ghana [18] and other among a small convenience sample of female sex workers (FSWs) and MSM in Peru [19]. The latter studies reported side-effects, efficacy and cost as important attributes, with good overall acceptability of PrEP. Hitherto, comparable data on acceptability of oral and parenteral PrEP medication, as well as key features of potential implementation programs, among different potential user groups and across countries, is largely missing.

In this research, we explored the attitudes and preferences towards hypothetical and known attributes of PrEP programs and medications (oral and parenteral), and ultimately, the future acceptability of PrEP, among five key populations in seven countries: Peru (FSWs and MSM), Ukraine (FSWs and injecting drug users (IDUs)), Kenya (HIV negative partners of heterosexual serodiscordant couples (SDCs) and FSWs), Uganda (SDCs and young women), Botswana (SDCs and young women), South Africa (young women and MSM) and India (FSWs and MSM). We aimed to better understand heterogeneity in attitudes and considerations about the regimen that would influence those attitudes.

## Methods

### Data Collection

Between October 2010 and May 2011, we administered a questionnaire to individuals from five key populations in seven countries to assess their likelihood of adopting PrEP. To ensure consistency in the quality of the data collection, we commissioned the international market research company Ipsos MORI to coordinate and supervise the fieldwork, and experienced local market research companies to carry it out. Fieldworkers had previous experience interviewing these populations and were trained face-to-face by researchers from Imperial College and/or Ipsos MORI. Individual questionnaire items were discussed with local researchers in a focus group setting to check pertinence and clarity of wording.

We piloted the study in Kenya (132 FSWs and 131 SDCs) and conducted 11 cognitive interviews in India (five MSM, three male sex workers, and three FSWs) to test questionnaire items' understandability and content validity. Questionnaires were translated in 16 languages by the local market research teams and back-translated by professional translators in London for content consistency. The final translation was agreed by consensus. Questionnaires were administered in the participants' native language. All participants completed the anonymous 20 minutes questionnaire and were offered a monetary incentive, except in South Africa, as required by its ethical committee.

The protocol of this study was approved by the ethical committee of Imperial College London; Health Research and Development Division, Ministry of Health (Botswana); Independent Ethics Committee (IEC), Bangalore (India); Kenya Medical Research Institute (KEMRI); Comite Institucional de Etica (CIE), Universidad Peruana Cayetano Heredia (Peru); Human Research Ethics Committee (Medical), University of Witwatersrand, Johannesburg (South Africa); Director General Health Services Ministry of Health (Uganda); and the Committee of Professional Ethics of the Sociological Association of Ukraine (SAU). We obtained informed written consent from all participants.

### Sample

We purposively chose countries with diverse HIV epidemics in different regions, selecting two potential user groups per country based on the predominant local modes of HIV transmission [20] and accessibility. We used targeted sampling [21] to recruit MSM, SDCs, FSWs and IDUs, and quota sampling to recruit young women [22]. We selected different geographical areas and a wide range of locations to ensure a diverse sample. Recruitment locations included: hairdressing salons, healthcare centers, hotels, non-governmental organizations (NGOs), nightclubs, red-light districts, saunas, streets, and universities for MSM; antiretroviral and community centers, dispensaries, healthcare centers, family planning clinics, and NGOs for SDCs; healthcare centers, hotels, NGOs, nightclubs, red-light districts, saunas and streets for FSWs; bars, churches, clinics, estates, health centers, homes, kiosks, markets, restaurants, salons, shops, streets and YMCA for young women; and needle-exchange points and NGOs for IDUs. Eligibility was determined using a brief screening interview where inclusion criteria were being identified as belonging to the relevant group, an age of 18 (16 for young women in Botswana) or more, self-reporting a negative or unknown HIV serostatus, being sexually active, and not having taken part in a market research study in the past 12 months.

### Measurement

We used a combination of quantitative measures (sections 1–3 and section 5) and conjoint analysis (section 4). The questionnaire had a total of 57 items in five sections. The first four sections were interviewer-administered, while section five was self-administered [23]. We employed verbal labels to improve data quality [24]. Unless specified here, we used four-point Likert scale items (1="yes, definitely”, 2="yes, probably”, 3="no, probably not”, and 4="no, definitely not”) to avoid midpoints, which can discourage respondents from taking a stand [25]. However, interviewers were allowed to record spontaneous “I do not know” responses.

Section one introduced PrEP as a medication which would reduce the risk of HIV infection in HIV negative people. A description of hypothetical and known PrEP attributes, constructed through expert consultations and a literature review [26], was provided. Participants were told that PrEP was ineffective against other sexually transmitted diseases, that was being tested as a pill and eventually as an injection [27], which could cause mild temporary side effects such as tiredness, headaches and gassiness, and that could be partially protective against HIV, especially if not taken as directed, therefore frequent HIV tests would be needed. It was stressed that PrEP was still being tested and its attributes remained uncertain. Participants were encouraged to ask the interviewer to repeat the description if any part was unclear. Questions about adherence to previous regular medication regimes were asked towards the beginning of this section, as a proxy measure for future adherence [28].

Section two explored the future acceptability and potential use of PrEP. We examined participants' willingness to take PrEP, likelihood of early adoption, and key feelings associated with taking PrEP: embarrassment, anxiety, hope, and fear of contracting HIV.

In section three, we assessed potential barriers to PrEP use: side effects, cost (an affordable and comparable monthly amount equivalent to two boxes of headache tablets in local currency, as condoms are often free of charge), willingness to share and sell it if given for free (a limited amount for personal use), condom use, and HIV testing.

In section four, we elicited data for conjoint analysis, a statistical technique frequently used to determine the value people assign to different features of products or services [29], [30] to assess the relative importance of key hypothetical and known attributes of PrEP. We chose attributes that represented relevant stages of a realistic implementation program, based on discussions with academic, policy, and industry experts. Conjoint analysis was conducted as follows. First, participants were shown a card with three different PrEP scenarios depicted on it, using both graphics and text to reduce cognitive effort. Each scenario had a different combination of five attributes (and corresponding levels): (1) route of administration (a pill once a day, a pill before and after having sex, an injection in the arm once a month, or an injection in the buttocks every two months); (2) dispensing site (pharmacy, family planning clinic, health clinic, or ARV clinic (NGOs in the case of Peru)); (3) time spent obtaining PrEP (two hours and four hours); (4) frequency of pick up (every month and every two months); and (5) Frequency of HIV testing associated with PrEP (monthly or every six months). Participants then indicated their preferred choice among the three different PrEP scenarios depicted on each card, with the option to state that none of the scenarios was preferable. Each participant responded to ten different cards.

Section five collected demographic data, including gender, place of residence, age, and education, which we used as proxy measure for socioeconomic status [31]. Participants were then asked to disclose sensitive information to assess risk behaviors, including number of sexual partners, type of sex practiced (vaginal and anal), HIV status, condom, and drug use. Before commencing this section, participants were reminded about the strict confidentiality of their responses. Subsequently, they were given a booklet with pictorial representations of the answers to facilitate comprehension [32]. We adapted a voting box approach to reduce social desirability bias [33] and asked participants to introduce the filled-out booklet in a blank envelop, seal it, and place it into a larger envelope containing other sealed booklets. Booklets had a unique code to link them back to the interviewer-administered part of the questionnaire.

### Statistical analysis

Analyses of variance (ANOVA) were conducted to examine the variability of participants' responses within and across countries. Tukey test results confirmed equal variances between the different groups across countries. Spearman's rank test was used to determine correlations between questionnaire items. Conjoint analysis was used to examine the relative importance of key attributes of PrEP. Five attributes were used to represent PrEP scenarios. To reduce cognitive effort we combined “time spent obtaining PrEP” and “frequency of pick up”, yielding 128 possible scenarios. An efficient design of 32 scenarios was found and 120 choice tasks were generated from these 32 scenarios (by combining scenarios together into sets of three) using SAS 9.3 software. Finally, the 120 choice tasks were split into twelve blocks of ten choice tasks. Sawtooth CBC/HB Version 5.2.8 software was used to decant respondents' choices into respondent-level utilities, using hierarchical Bayes estimation, which allowed us to determine the directionality (positive versus negative) and relative importance of each level. Due to the inherent heterogeneity of the sample, estimation of the utility scores was performed using different models for each user group and country. Therefore, levels' scores should not be compared.

## Results

### Participant characteristics

We interviewed a total of 1,824 participants and excluded from the sample 34 participants who self-reported a positive HIV serostatus, leaving a total sample of 1,790. As shown in Table 1, the majority of participants was female (61%), between 16 and 24 years of age (42%), had completed secondary or post secondary education (64%) and were black (49%). Most respondents reported between one and five sexual partners in the last month, having vaginal sex several times a week in the last year (46%), not having anal sex in the last year (54%), using condoms all the time in the last month (48%), not engaging in transactional sex at present (56%), not using injected drugs (87%) and not injecting drugs with a re-used needle in the past month (94%), as reported in Table 2.

**Table 1:** Characteristics of participants.

	Key populations at higher risk					
Characteristic	MSM	SDCs	FSWs	YW	IDUs	Total – n (%)
	N = 383^a^	N = 386	N = 514	N = 379	N = 128	
**Gender – n (%)**						
Male	361 (94)	209 (54)	NA	NA	99 (77)	669 (37)
Female	NA	176 (46)	514 (100)	379 (100)	29 (23)	1098 (61)
Transgender	22 (6)	NA	NA	NA	NA	22 (1)
Not stated	NA	1 (0)	NA	NA	NA	1 (0)
**Age group – n (%)**						
16–24 yr	150 (39)	39 (10)	168 (33)	377 (99)	22 (17)	756 (42)
25–30 yr	118 (31)	138 (36)	158 (31)	NA	38 (30)	452 (25)
31–40 yr	91 (24)	160 (41)	137 (27)	NA	45 (35)	433 (24)
≥41 yr	24 (6)	49 (13)	51 (10)	2 (1)	23 (18)	149 (8)
**Education level – n (%)**						
Less than secondary	88 (23)	176 (46)	186 (36)	153 (40)	26 (20)	629 (35)
Completed secondary	141 (37)	100 (26)	194 (38)	151 (40)	73 (57)	659 (37)
Postsecondary	152 (40)	105 (27)	128 (25)	71 (19)	29 (23)	485 (27)
Rather not say/not stated	2 (1)	5 (1)	6 (1)	4 (1)	-	17 (1)
**Race or ethnic group – n (%)**						
Black	51 (13)	386 (100)	129 (25)	315 (83)	NA	881 (49)
Mixed race	27 (7)	NA	NA	25 (7)	NA	52 (3)
White	22 (6)	NA	130 (25)	21 (6)	128 (100)	301 (17)
Asian Indian	154 (40)	NA	130 (25)	18 (5)	NA	302 (17)
Hispanic	129 (34)	NA	125 (24)	NA	NA	254 (14)
**Country where interview took place – n (%)**						
Peru^b^	129 (34)	NA	125 (24)	NA	NA	254 (14)
Ukraine^c^	NA	NA	130 (25)	NA	128 (100)	258 (14)
India^d^	128 (33)	NA	130 (25)	NA	NA	258 (14)
Kenya^e^	NA	127 (33)	129 (25)	NA	NA	256 (14)
Botswana^f^	NA	129 (33)	NA	129 (34)	NA	258 (14)
Uganda^g^	NA	130 (34)	NA	126 (33)	NA	256 (14)
South Africa^h^	126 (33)	NA	NA	124 (33)	NA	250 (14)

Percentages may not total 100 because of rounding. NA denotes not applicable, MSM men who have sex with other men, SDCs serodiscordant couples, FSWs female sex workers, YW young women, IDUs injection drug users, NGOs non governmental organisations and ARV antiretroviral.

a 20% of MSM were male sex workers. Interviews were conducted in:

b Lima and Callao.

c Donetsk, Kharkiv, Mykolayiv, and Vinnitsa.

d Bangalore, Chennai, Delhi, Hyderabad, Kolkata, Mumbai, Namakkal, and Pune.

e Kisumu, Mombasa, and Nairobi.

f Gabane, Gaborone, Kanye, Kweneng, Lobatse, Metsimotlhabe, Mochudi, Ramotswa, and Tlokweng.

g Jinja, Kampala, and Mbarara.

h Bloemfontein, Cape Town, East London, Durban, Johannesburg, Kimberley, Mafikeng, Nelspruit, and Polokwane.

**Table 2:** Characteristics of participants – risk factors.

	Key populations at higher risk					
Characteristic	MSM	SDCs	FSWs	YW	IDUs	Total – n (%)
	N = 383	N = 386	N = 514	N = 379	N = 128	
**Sexual risk factors** a						
Number of partners in the last month – n (%)						
0	9 (2)	12 (3)	2 (0)	17 (4)	8 (6)	48 (3)
1–5 partners	264 (69)	355 (92)	111 (22)	264 (70)	111 (88)	1105 (62)
6–10 partners	57 (15)	4 (1)	79 (15)	11 (3)	-	151 (8)
11–20 partners	27 (7)	1 (0)	108 (21)	8 (2)	-	144 (8)
≥21 partners	17 (4)	-	213 (41)	1 (0)	1 (1)	232 (13)
Not stated	9 (2)	14 (4)	1 (0)	91 (24)	8 (6)	123 (7)
Frequency of vaginal sex in the last year^b^ – n (%)						
Several times a week	53 (14)	176 (46)	428 (83)	98 (26)	68 (53)	823 (46)
About once a week	42 (11)	105 (27)	64 (12)	82 (22)	33 (26)	326 (18)
About once a month	26 (7)	51 (13)	12 (2)	60 (16)	12 (9)	161 (9)
Less often than once a month	39 (10)	29 (8)	8 (2)	30 (8)	10 (8)	116 (7)
Not at all	223 (58)	25 (6)	-	32 (8)	5 (4)	285 (16)
Not stated	-	-	2 (0)	77 (20)	-	79 (4)
Frequency of anal sex in the last year – n (%)						
Several times a week	186 (49)	7 (2)	57 (11)	11 (3)	-	261 (15)
About once a week	113 (30)	8 (2)	62 (12)	15 (4)	3 (2)	201 (11)
About once a month	40 (10)	4 (1)	67 (13)	13 (3)	12 (9)	136 (8)
Less often than once a month	29 (8)	11 (3)	55 (11)	27 (7)	13 (10)	135 (8)
Not at all	-	353 (91)	273 (53)	236 (62)	100 (78)	962 (54)
Not stated	15 (4)	3 (1)	-	77 (20)	-	95 (5)
Frequency of condom use in the last month – n (%)						
All the time	199 (52)	214 (55)	319 (62)	102 (27)	20 (16)	854 (48)
Most of the time	96 (25)	88 (23)	127 (25)	60 (16)	34 (27)	405 (23)
Some of the time	45 (12)	35 (9)	39 (8)	53 (14)	17 (13)	189 (11)
Rarely	11 (3)	12 (3)	16 (3)	14 (4)	10 (8)	63 (4)
None of the time	13 (3)	11 (3)	10 (2)	39 (10)	31 (24)	104 (6)
Not stated	19 (5)	26 (7)	3 (1)	11 (3)	16 (13)	75 (4)
Transactional sex at present – n (%)						
Yes	164 (43)	45 (12)	514 (100)	63 (17)	-	786 (44)
No	219 (57)	341 (88)	-	316 (83)	128 (100)	1004 (56)
**Injecting drug use risk factors**						
Injecting drugs at present – n (%)						
Yes	25 (7)	12 (3)	54 (11)	12 (3)	128 (100)	231 (13)
No	357 (93)	374 (97)	456 (89)	367 (97)	-	1554 (87)
Not stated	1 (0)	-	4 (1)	-	-	5 (0)
Injected drugs with re-used needle in past month – n (%)						
0	361 (94)	379 (98)	483 (94)	369 (97)	92 (72)	1684 (94)
1–5 times	17 (4)	4 (1)	29 (6)	8 (2)	33 (26)	91 (5)
6–10 times	2 (1)	2 (1)	1 (0)	1 (0)	2 (2)	8 (0)
≥11 times	1 (0)	-	-	-	1 (1)	2 (0)
Not stated	2 (1)	1 (0)	1 (0)	1 (0)	-	5 (0)

Percentages may not total 100 because of rounding. MSM men who have sex with other men, SDCs serodiscordant couples, FSWs female sex workers, YW young women and IDUs injection drug users.

a “Not stated” in this section includes participants who reported never having had sex.

b Vaginal sex reported by MSM was bisexual.

### Future acceptability and potential use of PrEP

**Figure 1:**
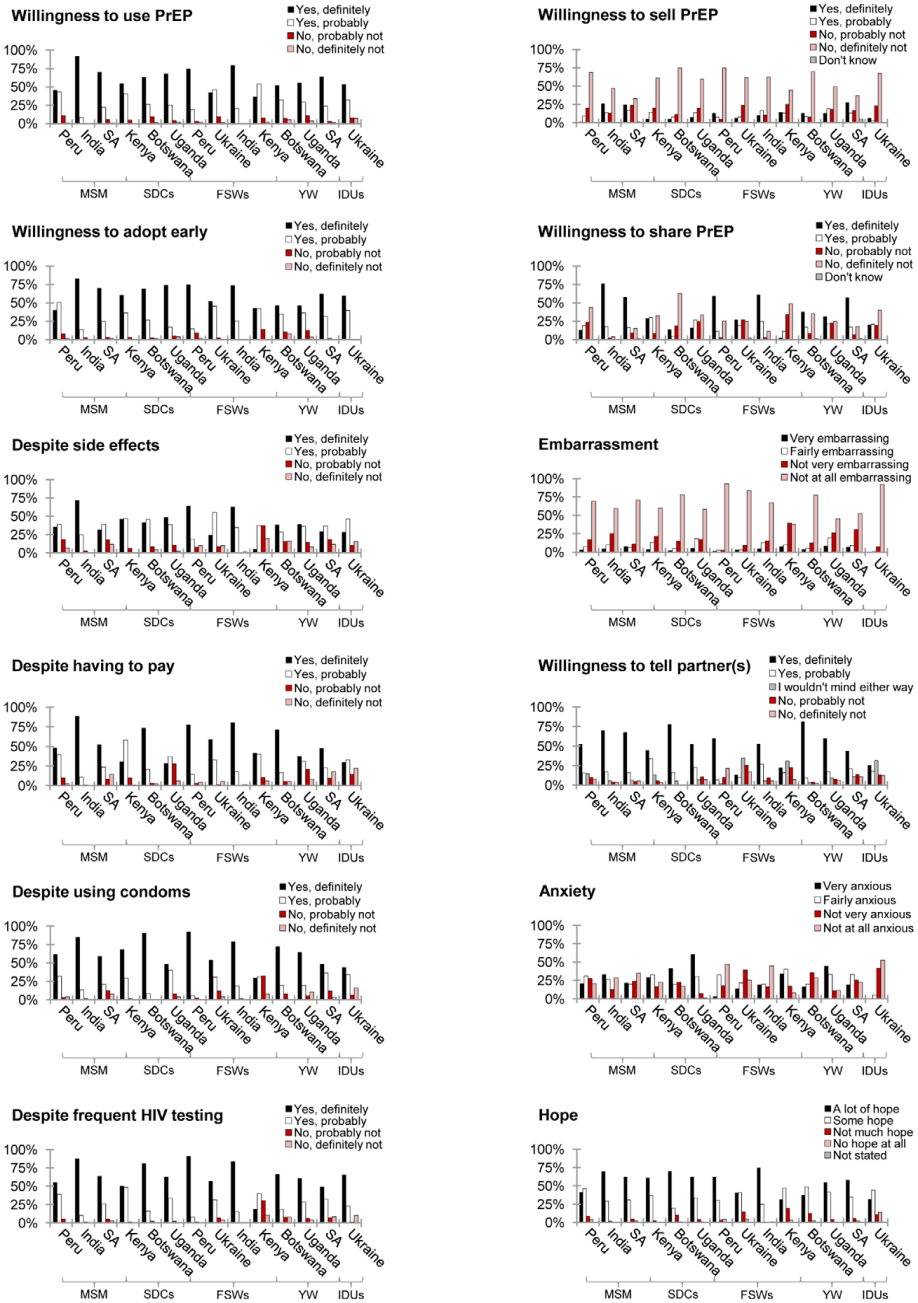
Acceptance of PrEP. SDCs denotes serodiscordant couples, MSM men who have sex with other men, FSWs female sex workers, YW young women and IDUs injection drug users.

As reported in Figure 1, participants were generally willing to use PrEP (61% “yes, definitely” and 30% “yes, probably”) and to adopt it early, i.e. “as soon as it becomes available” (61% “yes, definitely” and 31% “yes, probably”). Participants indicated willingness to use PrEP despite potential side effects (40% “yes, definitely” and 38% “yes, probably”), and even if they had to pay for it (55% “yes, definitely” and 29% “yes, probably”), use a condom in combination with PrEP (64% “yes, definitely” and 24% “yes, probably”), or be regularly tested for HIV (64% “yes, definitely” and 27% “yes, probably”). Participants showed little interest in selling PrEP (12% “yes, definitely” and 12% “yes, probably”), but reported intentions to share it (36% “yes, definitely” and 18% “yes, probably”). As shown in Table 3, FSWs in Kenya were less inclined to use PrEP in the presence of side effects than participants in other groups and countries (M = 2.73, p<.05). IDUs in Ukraine (M = 1.95, p<.05) and FSWs in Kenya (M = 2.17, p<.05) were less willing to accept PrEP in combination with a condom than participants in other groups and countries. FSWs in Kenya were also less likely to accept PrEP than participants in other groups and countries if they had to be regularly tested for HIV (M = 2.33, p<.05).

**Table 3:** Analysis of variance of attitudes and preferences towards PrEP.

Question	Country and key population													
	India	India	Peru	Peru	Ukraine	Ukraine	Kenya	Kenya	Uganda	Uganda	Botswana	Botswana	SA	SA
	FSWs	MSM	FSWs	MSM	IDUs	FSWs	SDCs	FSWs	SDCs	YW	SDCs	YW	MSM	YW
If PrEP becomes available, do you think you would use it?	1.21^de^	1.08^e^	1.35^cde^	1.67^abc^	1.68^ab^	1.72^ab^	1.50^abcd^	1.76^a^	1.41^bcd^	1.65^abc^	1.49^abcd^	1.75^a^	1.42^bcd^	1.65^abc^
Would you take PrEP as soon as it becomes available, or not?	1.28^bc^	1.20^c^	1.38^bc^	1.71^ab^	1.41^bc^	1.50^ab^	1.43^bc^	1.73^a^	1.39^bc^	1.76^a^	1.37^bc^	1.81^a^	1.37^bc^	1.55^ab^
Would you take PrEP if it caused mild temporary side effects, or not?	1.41^e^	1.33^e^	1.64^de^	1.97^bcd^	2.12^bc^	2.08^bc^	1.62^de^	2.73^a^	1.67^de^	1.97^bcd^	1.76^cde^	2.14^b^	2.09^bc^	2.24^b^
Would you take PrEP if you had to pay a month for it, or not?	1.22^ef^	1.12^f^	1.35^def^	1.66^cd^	2.29^a^	1.55^cde^	1.81^bc^	1.86^bc^	2.11^ab^	2.05^ab^	1.34^def^	1.47^de^	1.88^bc^	2.04^ab^
Would you take PrEP even if you have to use condoms, or not?	1.24^fg^	1.16^g^	1.10^g^	1.49^cdef^	1.95^ab^	1.65^bcde^	1.35^efg^	2.17^a^	1.67^bcd^	1.62^cde^	1.10^g^	1.37^defg^	1.68^bc^	1.72^bc^
Would you take PrEP if you needed to be tested regularly for HIV, or not?	1.17^e^	1.15^e^	1.11^e^	1.50^cd^	1.56^bc^	1.60^bc^	1.52^cd^	2.33^a^	1.41^cde^	1.55^bc^	1.23^de^	1.57^bc^	1.52^cd^	1.84^b^
How embarrassing, if at all, would you find it to take PrEP?	3.45^bcd^	3.42^bcd^	3.89^a^	3.62^abc^	3.92^a^	3.76^ab^	3.42^bcd^	3.23^d^	3.31^cd^	3.11^d^	3.69^ab^	3.66^ab^	3.58^abc^	3.31^cd^
How anxious, if at all, does the thought of taking PrEP make you feel?	2.86^bc^	2.36^defg^	3.08^ab^	2.48^cdef^	3.45^a^	2.76^bcd^	2.32^efgh^	2.00^gh^	1.50^i^	1.89^hi^	2.15^fgh^	2.76^bcd^	2.72^bcde^	2.51^cdef^
Would you want your partner or partners to know if you were taking PrEP, or not?	1.87^cde^	1.55^ef^	2.26^bc^	2.05^cd^	2.69^b^	3.23^a^	1.88^cde^	2.77^ab^	1.98^cde^	1.83^cdef^	1.29^efg^	1.36^ef^	1.64^defg^	2.27^bc^
How much hope, if any, does PrEP give you for new possibilities for you in life?	1.28^e^	1.32^e^	1.50^de^	1.75^bcd^	2.07^a^	1.84^ab^	1.42^e^	1.94^ab^	1.43^e^	1.49^de^	1.42^e^	1.80^abc^	1.47^de^	1.52^cde^
Would you share PrEP with other people who need it more than you, or not?	1.65^ef^	1.34^f^	1.97^de^	2.98^ab^	2.79^bc^	2.56^bc^	2.47^c^	3.40^a^	2.75^bc^	2.40^cd^	3.32^a^	2.46^c^	1.87^e^	1.90^e^
Would you sell PrEP to other people who need it more than you, or not?	3.25^abc^	2.81^d^	3.41^abc^	3.58^a^	3.52^ab^	3.43^abc^	3.36^abc^	3.09^bcd^	3.30^abc^	3.04^cd^	3.58^a^	3.37^abc^	2.70^d^	2.79^d^

Means with different subscripts are significantly different, *p*<.05. MSM men who have sex with other men, SDCs serodiscordant couples, FSWs female sex workers, YW young women and IDUs injection drug users. Lower means represent a more positive response.

Our findings also show that participants' levels of embarrassment associated with taking PrEP were generally low (4% “very embarrassing” and 9% “fairly embarrassing”) and that they would want their partner or partners to know they were taking it (52% “yes, definitely” and 18% “yes, probably”) (Figure 1). However, the thought of taking PrEP made participants feel anxious (26% “very anxious” and 26% “fairly anxious), particularly in the case of SDCs in Uganda (M = 1.50, p<.05) (Table 3). Nonetheless, participants generally felt that PrEP would give them hope for new possibilities in their lives (54% “a lot of hope” and 36% “some hope”) (Figure 1).

### Participants' characteristics and likelihood of PrEP use

Spearman's rank correlations in Table 4 show that participants reporting adherence to past medication (r = .10, p<.01), female participants (r = .05, p<.05), participants of younger age (r = .08, p<.01), participants with fewer children (r = .10, p<.01), higher condom usage in the last month (r = .11, p<.01), participants who tested for HIV in the past (r = .10, p<.01), never injected drugs (r = .12, p<.01), and currently do not inject drugs (r = .09, p<.01), were more likely to use PrEP in general. We found no significant correlation between likelihood of PrEP use and frequency and type of exposure (anal vs. vaginal), and education.

**Table 4:** Participants' characteristics and likelihood of PrEP use.

	1	2	3	4	5	6	7	8	9
1. Willingness to use PrEP	1.00								
2. Adherence to previous medicine	.10**	1.00							
3. Gender	.05*	.04	1.00						
4. Age	−.08**	−.04	−.26**	1.00					
5. Number of children	−.10**	−.02	.04	.52**	1.00				
6. Condom usage	.11**	.10**	−.03	.10**	−.02	1.00			
7. Tested for HIV/AIDS	.10**	.04	.08**	.29**	−.23**	.13**	1.00		
8. Ever injected drugs before	−.12**	−.02	.10**	.05*	.05*	−.15**	.06**	1.00	
9. Currently injecting drugs	−.09**	.03	.13**	.05*	.06*	−.18**	.09**	.76**	1.00

** Correlation significant at .01 level.

*Correlation significant at .05 level (2-tailed).

Numbers in the column headings represent the characteristics enumerated in the row headings.

### Relative importance of PrEP attributes

**Figure 2:**
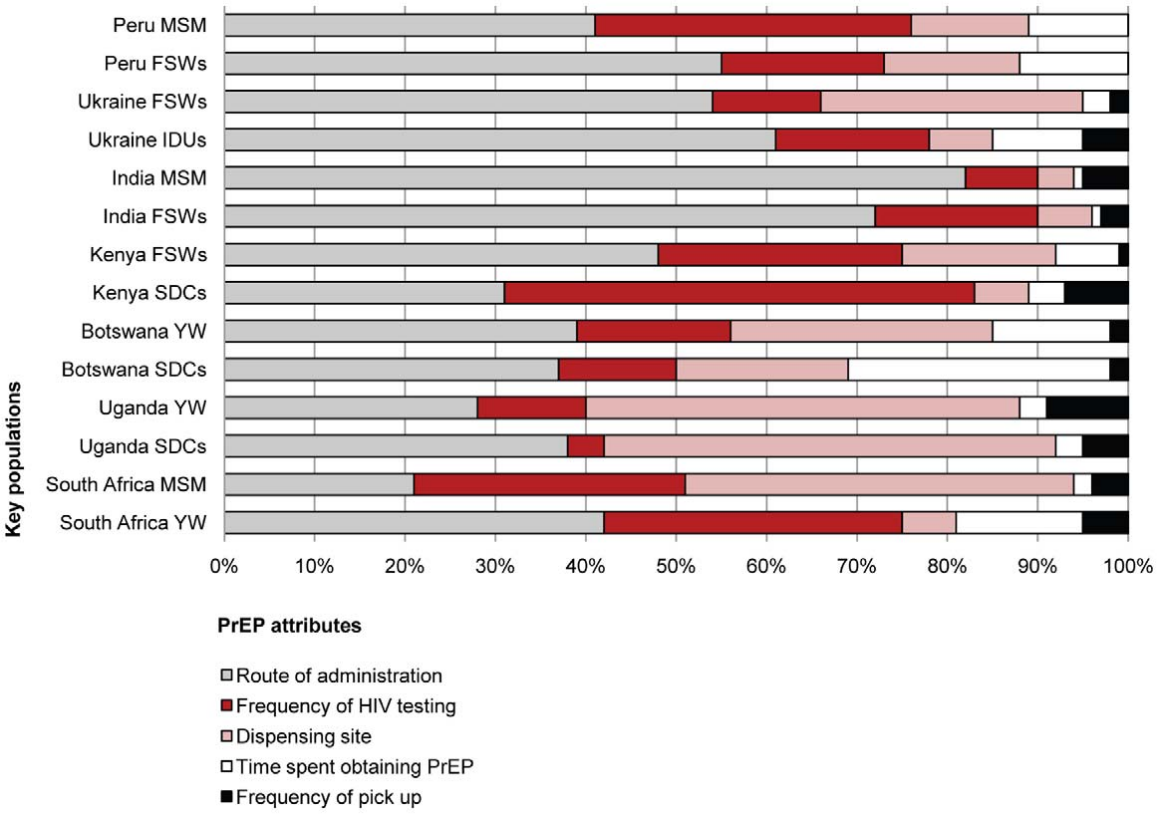
Relative importance of key PrEP attributes. SDCs denotes serodiscordant couples, MSM men who have sex with other men, FSWs female sex workers, YW young women and IDUs injection drug users.

Results in Figure 2 show the relative importance of five attributes of PrEP by key group and country. The route of administration was the most important attribute for Peruvian, Ukrainian, Indian and Batswana participants, and FSWs in Kenya and young women in South Africa. PrEP dispensing site, on the other hand, was the most important attribute for Ugandan participants and MSM in South Africa, and the second most important attribute for FSWs in Ukraine. HIV testing was the second most important attribute for Peruvian, Indian and Kenyan participants, and IDUs in Ukraine and young women in South Africa. Time spent obtaining PrEP and frequency of pickup were generally less important.

**Figure 3:**
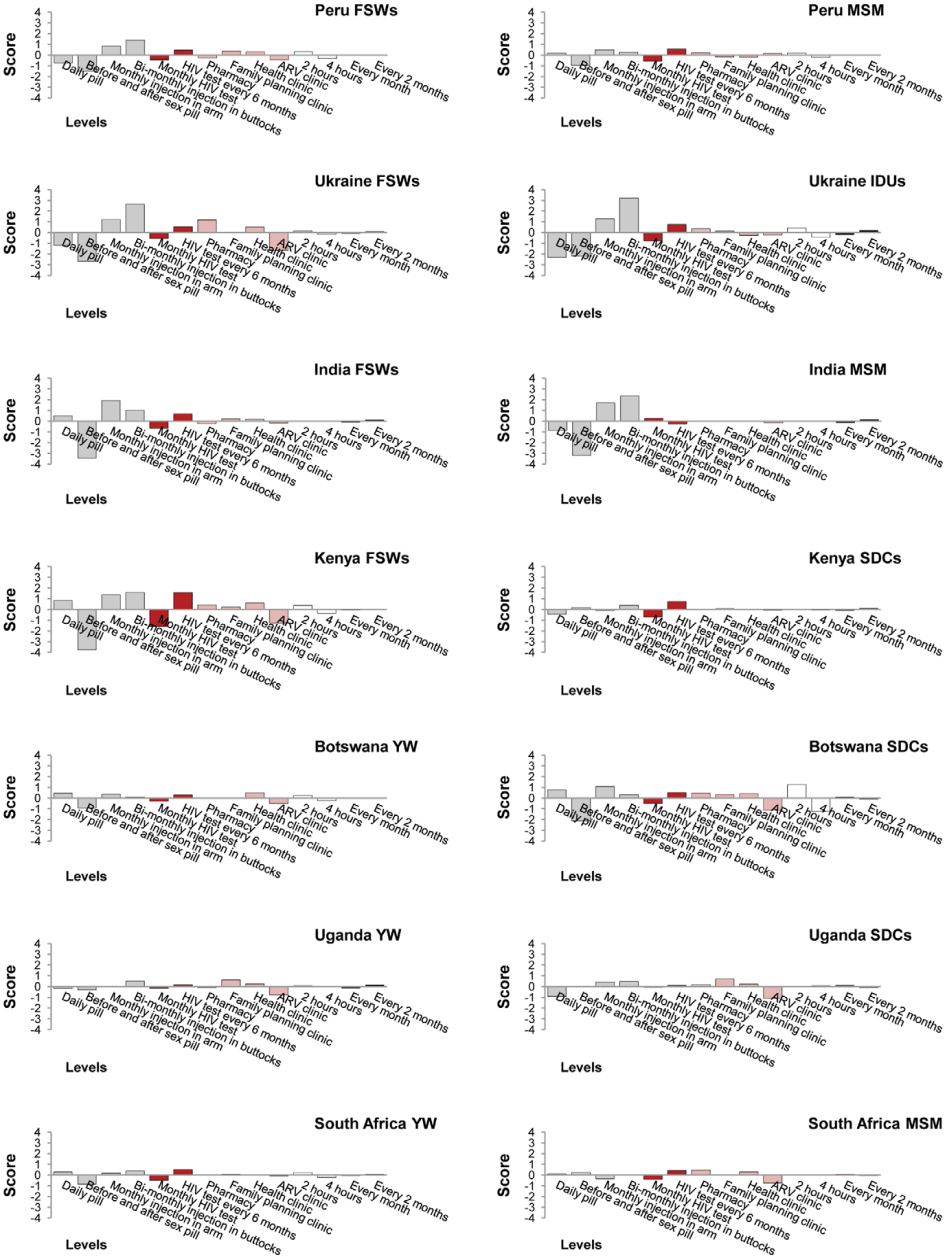
Marginal utilities—relative importance of the levels of PrEP attributes. SDCs denotes serodiscordant couples, MSM men who have sex with other men, FSWs female sex workers, YW young women and IDUs injection drug users. (4, −4) is an arbitrary interval.

Figure 3 shows participants' preferences regarding the different alternatives of each PrEP attribute. A bimonthly injection in the buttocks was the most preferred alternative of the route of administration, followed by a monthly injection in the arm, while a daily pill and a pill before and after sex were the least preferred options. The most preferred HIV testing frequency is every six months as opposed to monthly. Results regarding dispensing sites were heterogeneous, with the exception of ARV clinics, which was the least preferred alternative. Time spent obtaining PrEP and frequency of pick up were not influential determinants of PrEP use for most participants.

## Discussion

We estimated the future acceptability of PrEP, examining the attitudes and preferences of potential user groups from different countries towards hypothetical and known PrEP attributes. Our results show that participants were generally willing to accept PrEP and adopt it as soon as it becomes available. Surprisingly, participants were also willing to take PrEP even when reminded of potential side effects, cost, condom use, and frequent HIV testing. These findings indicate participants' motivation to overcome barriers which can have a considerable impact on uptake. In contrast, participants mentioned that the thought of taking PrEP made them feel anxious, although they also indicated that taking PrEP would not be embarrassing and they would want their partner or partners to know. Participants' anxiety may be explained by the hypothetical nature of most of the presented PrEP characteristics, the stigma associated with HIV [34], and in some settings, the criminalization of sex work, injected drug use and homosexuality [35]. Most participants, nonetheless, subsequently indicated that PrEP would give them hope, which suggests that their initial willingness to take it remained largely unscathed.

Female participants indicated a higher level of willingness to take PrEP than male participants, which may be explained by women's difficulty negotiating the use of condoms and awareness of their and/or their partners' risk of becoming infected with HIV [36]. We also found that younger participants and those with fewer children, those who reported adherence to past medication, more frequent condom usage, having been tested for HIV in the past and never injecting drugs, reported greater willingness to take PrEP. These promising findings suggest that those who are currently bearing the brunt of HIV [1], have higher perceived risk, and are most likely to adhere to a comprehensive PrEP program, are also the most motivated to enroll. Yet, while participants stated not being interested in selling PrEP, the majority reported intentions to share it. Therefore, information and counseling about the risks of sharing PrEP should be readily available as part of any implementation program.

Results from the conjoint analysis reveal trends in participants' preferences which deserve consideration. PrEP route of administration was the most important attribute, and bi-monthly and monthly injections were the preferred alternatives. This finding is encouraging from a policy perspective if such modalities become available; since it may reduce users' likelihood of sharing, selling or forgetting to take PrEP, but it also raises questions regarding participants' willingness to take oral PrEP. HIV testing was the second most important attribute, and a test every six months was, as expected, the preferred alternative. Interestingly, dispensing sites were more important than any other attribute for some groups, particularly in Africa. This may indicate concerns about social stigma and access [37]. However, it is encouraging that most participants were willing to receive PrEP at a healthcare facility, which can facilitate synergies between PrEP and other existing prevention services. Time spent obtaining PrEP and frequency of pick up, which we used as a proxy measure for cost-opportunity, were generally less important, consistent with participants' willingness to pay for PrEP.

Our findings are broadly consistent with the work of Guest et al. and Galea et al [18], [19]. However, specific comparisons are not advisable as the composition and size of the samples, recruitment methods, measures and statistical analyses differ greatly. Previous work on PrEP implementation suggests that delivery programs will need to meet a number of requirements in order to be effective, including: prioritization of groups at higher risk of infection; delivery of PrEP in combination with other prevention services, including risk reduction and medication adherence counseling, condoms provision, diagnosis and treatment of other sexually transmitted infections, and frequent HIV testing; and monitoring of side effects, adherence and risk behaviors [8], [38], [39], [40], [41], [42]. Our results provide valuable clues that can help countries to deliver PrEP more effectively, should they decide to implement it, by focusing their efforts on the aspects that need more attention.

This is the first multinational study, to our knowledge, that integrates different disciplines to shed light on a question that we believe is of global importance. Our study complements previous work on PrEP by examining potential users' perspective and offering insights into their attitudes and preferences. We note that it may not be possible to generalize the observed PrEP acceptability to other settings and our results should be considered within the context of this study's limitations. Given the sensitive nature of the addressed questions, and despite all our efforts to reduce social desirability bias, there is an unavoidable risk that participants may have felt at times compelled to provide what they felt was the “right” answer. Additionally, our data collection took place in urban areas, where HIV incidence is normally higher, thus current findings may not be generalizable to rural settings. Finally, examining acceptability among users enrolled in pilot programs is much deserving, as actual acceptability may differ from potential willingness to take PrEP, especially if relevant attributes of a product or program are modified, as observed in other comparable interventions [43].

### Conclusions and recommendations

We have shown that key populations across different countries would be willing to take PrEP despite multiple barriers and uncertainty. Our findings suggest that those who are most at risk of infection are ready to adopt alternative HIV prevention methods, and PrEP appears to be an acceptable one. Adherence, risk compensation and inappropriate use are legitimate concerns, as it is the cost and complexity of rolling out and integrating PrEP into combination prevention packages. However, significantly reducing the burden of the epidemic, especially in high incidence settings, will only be possible if existing prevention efforts are strengthened and expanded, and innovative approaches are introduced.

Our results suggest that an effective PrEP implementation strategy should be country-specific, but they also show common trends which are worth highlighting. Communicating PrEP benefits and disadvantages in a transparent, unbiased and concise manner will help to dissipate users' anxieties and facilitate uptake. Offering PrEP at different healthcare facilities would be acceptable for users and recommendable from a policy perspective. Asking for a copayment within a cost-segmented strategy should be considered, as an affordable amount will not only alleviate some of the financial burden on the public purse, but it could also increase the perceived value of PrEP, and therefore improve adherence. A “contract” between the user and the provider subject to adequate regimen adherence, which could be monitored by randomly measuring blood levels, may be advisable.

Introduction of new technologies should consider population specific preferences and concerns of potential users, which can be explored using pre-marketing research.
